# Overexpression of Spondin-2 Is Associated with Recurrence-Free Survival in Patients with Localized Clear Cell Renal Cell Carcinoma

**DOI:** 10.1155/2020/5074239

**Published:** 2020-09-03

**Authors:** Hui-Min Ma, Meng Yu, Cong Wu, Hou-Bao Huang, Ya-Wei Li, Peng Zhang, Jian-Jun Huang, Long Cheng, Gang Feng, Guo-Rong Li

**Affiliations:** ^1^Clinical Laboratory, The First Affiliated Hospital of Wannan Medical College, Wuhu, 241001 Anhui, China; ^2^Key Laboratory of Noncoding RNA Transformation Research of Anhui Higher Education Institution (Wannan Medical College), Wuhu, 241002 Anhui, China; ^3^Department of Laboratory Diagnosis, Changhai Hospital Navy Medical University, 200433 Shanghai, China; ^4^Department of Urology, The First Affiliated Hospital of Wannan Medical College, Wuhu, 241001 Anhui, China; ^5^Department of Urology, North Hospital, CHU of Saint-Etienne, Saint-Etienne 42055, France

## Abstract

**Background:**

The spondin-2 (SPON2) gene is overexpressed in multiple malignant tumors and may promote tumor aggressiveness. However, its expression profile and functional roles in clear cell renal cell carcinoma (ccRCC) are still unclear.

**Methods:**

SPON2 expression in ccRCC was evaluated using expression data from TCGA and GEO databases, then confirmed by local patient population (94 patients). The clinical significance of SPON2 expression was evaluated. Downregulation of SPON2 was performed using small-interfering RNA (siRNA). The effects of SPON2 silencing on cell proliferation, apoptosis, invasion, and migration *in vitro* were investigated.

**Results:**

SPON2 was overexpressed in the majority of the ccRCC at both mRNA and protein levels. SPON2 expression was significantly correlated with stage, grade, and recurrence (all *P* < 0.05) in patients with localized ccRCC. The receiver operating characteristic (ROC) curve showed that SPON2 expression could serve as a predictor of recurrence. SPON2 expression was significantly associated with recurrence-free survival (RFS) in patients with localized ccRCC. Knocking down SPON2 resulted in suppressed cell invasion and migration *in vitro*.

**Conclusion:**

SPON2 expression might function as a prognostic biomarker in patients with localized ccRCC.

## 1. Introduction

Clear cell renal cell carcinoma (ccRCC) is the most frequent histological type of kidney cancer, which has increasing incidence and mortality rates worldwide [1]. Although imaging examination is widely used to kidney cancer screening in recent years, about 30% of patients already have metastasis at the first diagnosis. While nephrectomy is a curative method for ccRCC, approximately 30% of patients will relapse during the course of disease [2, 3]. The VEGFR-TKI, mTOR inhibitors, and immune checkpoint inhibitors improve survival significantly; however, the prognosis of some advanced ccRCC patients remains poor [4, 5]. Therefore, it is urgent to discriminate high-risk patients who may be inclined to have a higher probability of recurrence.

The extracellular matrix (ECM) is composed of approximately 300 proteins that regulate tissue homeostasis, organ development, inflammation, and disease [6]. The dysregulated ECM including the loss of homeostasis and integrity, which has been observed in many different types of cancer and typically defined transitional events in progression and metastasis [7]. According to bioinformatics analysis based on data of ccRCC in the Cancer Genome Atlas (TCGA), many ECM genes had significantly abnormal expression in ccRCC tumors compared to controls. Among these dysregulated ECMs, overexpression of spondin-2 (SPON2) was statistically correlated with poor overall survival (OS) of patients with ccRCC. Previous studies showed that SPON2 expression was significantly associated with prognosis in hepatocellular carcinoma, colorectal cancer, gastric cancer, and lung adenocarcinoma [8–12].

However, SPON2 has not been studied about its expression, prognostic value, and functional roles in ccRCC. The purpose of this study was to investigate SPON2 expression in ccRCC and determine its association with clinicopathological characteristics and patient prognosis. Furthermore, functional analysis of SPON2 gene was also evaluated *in vitro*.

## 2. Materials and Methods

### 2.1. TCGA and Public Microarray Data Analysis

The RNA-sequencing data of TCGA dataset was available on the website of Gene Expression Profiling Interactive Analysis (GEPIA) [13]. Limma method was selected for differential analysis. The statistic significant criteria were set at the absolute log2 fold change (tumor/normal) ≥1.5 and *q* value ≤ 0.01 in the ccRCC database, respectively. Genes associated with patient survival also be investigated by GEPIA online tool. Three public RCC microarray gene profiling datasets (GSE53757 [14], GSE73731 [15], and GSE47352 [16]) were downloaded from the Gene Expression Omnibus (GEO). GSE53757 contained 72 paired ccRCC and normal adjacent tissues. GSE73731 contained 265 ccRCC including stages I-IV. GSE47352 contained 4 primary metastatic and 5 nonmetastatic ccRCC tumor samples. These RNA sequencing and microarray data were preprocessed by using R software and packages.

### 2.2. Patients and Samples

A total of 94 matched-pairs of ccRCC tissue (T) and adjacent normal tissue (N) were obtained from patients who underwent nephrectomy (radical or partial) without any neoadjuvant treatment between January 2018 and July 2019 at the First Affiliated Hospital of Wannan Medical College. All tumors were staged according to the 2017 AJCC TNM classification and graded according to the Fuhrman grading system by two senior pathologists. The information including age, gender, tumor diameter, TNM stage, Fuhrman grade, histological tumor necrosis, histological sarcomatoid, lymph node metastasis, distant metastasis, and recurrence were collected from each patient. Patient with localized ccRCC was defined as a patient with no evidence of distant metastasis or retroperitoneal lymph node metastasis on initial evaluation. Follow-up generally included physical examination, serum chemistry, liver function tests, chest radiography and abdominal ultrasonography, or CT every 6 months for the first year and annually thereafter. Recurrence-free survival (RFS) was calculated from the date of surgery to the date of recurrence (or the last follow-up). All procedures performed in studies involving human participants were reviewed and approved by the Ethics Committee of Wannan Medical College and with the 1964 Helsinki declaration and its later amendments or comparable ethical standards. Written informed consent was obtained from all individual participants included in the study.

### 2.3. Cell Lines and Culture

Human renal proximal tubule epithelial cell line HK-2, human ccRCC cell lines 786-O, and Caki-1 were cultured according to our previous description [17].

### 2.4. Protein Isolation and Western Blot Assay

Protein extraction of tissues or cells and western blot analysis were performed using a protocol published by Feng et al. [17]. Briefly, the membranes were incubated overnight with primary antibody against SPON2 (monoclonal #ab171955, Abcam) followed by HRP-conjugated secondary antibody.

### 2.5. Real-Time Quantitative PCR and Immunohistochemistry (IHC)

The protocols of real-time quantitative PCR and IHC were performed as described previously [17]. Briefly, PCR amplification was performed using gene-specific TaqMan Gene Expression Assays (SPON2: Hs00202813_m1; GAPDH: Hs99999905_m1). The relative expression level of SPON2 was calculated by normalization to GAPDH. Tissue sections were incubated with anti-SPON2 polyclonal antibody (#ab215451, Abcam) overnight followed by secondary antibody.

### 2.6. RNAi Knockdown

Three siRNA oligonucleotides (designated #1, #2, #3) and mismatch siRNA control oligonucleotides were synthesized by RiboBio Co. Ltd. (Guangzhou, China). The sequences of the best inhibited siRNA (designated #1) were as followed: siRNA-SPON2, 5′-GGAGGAAGAACCAGTACGT-3′; and siRNA-control, 5′-CAGAGGGAGUGGGAGCCAAUAAUUA-3′. The protocol of RNAi knockdown was performed as described previously [17].

### 2.7. Cell Proliferation and Apoptosis Assay

Cell proliferation was determined using real-time cellular analysis (RTCA). Twenty-four hours after transfection, cells (6 × 10^3^ cells/well) were seeded in several E-Plate 16 dishes (ACEA Biosciences Inc, CA, USA) for proliferation assays. The plates were kept in the cell incubator at 37°C with 5% CO_2_ for 6 days. The cell index and growth curves were automatically recorded on the xCELLigence RTCA System (ACEA Biosciences Inc, CA, USA). For colony formation assay, cells transfected with siRNAs were plated in 6-well plates at 700 cells/well then cultured for 2 weeks. The cells were fixed and stained for 30 min in 35% methanol solution with 1% crystal violet; then, a number of foci >100 cells were counted. For apoptosis analyses, Annexin V/PI staining of cells was carried out. At 48 hours after transfection, cells were harvested, washed, and stained with FITC Annexin V Apoptosis Detection kit (Beyotime, China). And then, apoptosis cells were determined by a flow cytometer (Guava easyCyte HT, Millipore). All assays were performed in triplicate.

### 2.8. Cell Migration and Invasion Assays

Cells were plated in 6-well plates and allowed to grow until 100% confluency. Then, cell layer was scratched through the central axis using a sterile plastic tip and loose cells were washed. The widths of the initial gaps (0 and 24 hours) were calculated using a microscope (Nikon Eclipse E200). Cell invasion was performed with a Costar transwell chambers (8-*μ*m pore size, 6.5 mm membrane diameter) were coated with 1 : 8 diluted matrigel (BD Biosciences, San Jose, CA, USA). A suspension of transfected cells (5 × 10^3^ cells) was placed in the upper chambers, and the lower chambers were filled with 500 *μ*L of RPMI 1640 containing 10% FBS. After 24 h of incubation, cells that had not invaded the pores were carefully wiped off with cotton swab. All migrated cells that remained on the bottom surface were fixed with 4% paraformaldehyde (Beyotime, Shanghai, China) for 30 min and stained with 0.1% crystal violet (Beyotime, Shanghai, China) for 10 min. Cells in five random fields of each chamber were counted and averaged. All assays were performed in triplicate.

### 2.9. Statistical Analyses

SPSS 19.0 version, GraphPad 5.0 software, and R programming language version 3.6.1 were used to perform statistical analysis in this study. Numerical data were analyzed by the Student *t*-test, Mann–Whitney *U* test, or Kruskal-Wallis test as appropriate. Categorical data were analyzed by a chi-squared test. Receiver operating characteristic (ROC) curve and the corresponding area under the curve (AUC) were calculated for testing the potential of SPON2 expression for prediction of recurrence after nephrectomy in patients with localized ccRCC. Survival curves were conducted using the Kaplan-Meier method, and the difference was analyzed by Log-rank test. Factors verified to have statistically significant prognostic value in a univariate Cox regression model were then entered into a multivariate Cox regression model. Harrell concordance index (C-index) and Akaike information criteria (AIC) analysis were applied to investigate the accuracy of each factor. Differences were regarded as statistically significant at *P* values (two-sided) of less than 0.05.

## 3. Results

### 3.1. SPON2 Is Overexpressed in ccRCC

**Figure 1:**
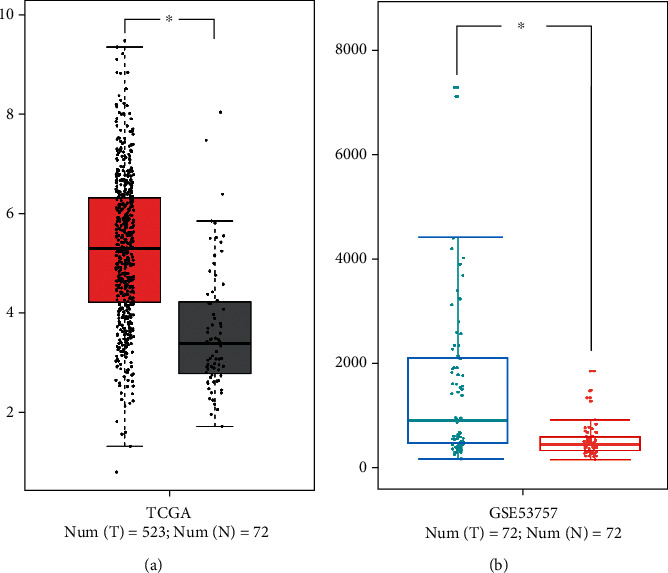
SPON2 expression in ccRCC and normal tissues. (a) TCGA ccRCC dataset. (b) GSE53757.

**Figure 2:**
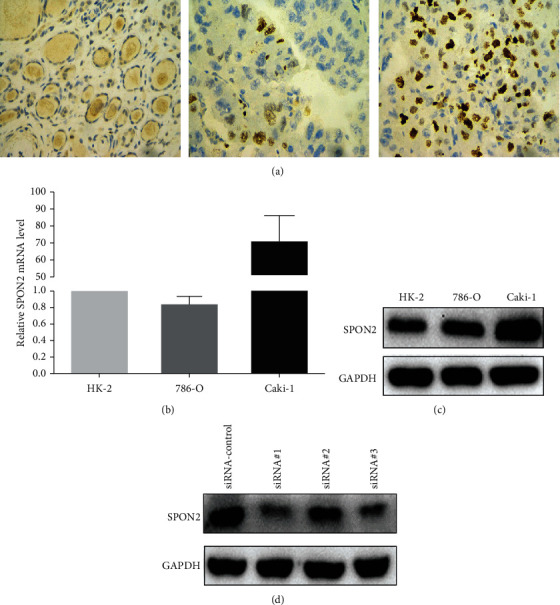
SPON2 expression in ccRCC and cell lines. (a) Representative SPON2 histologic scoring in ccRCC tissues. (b) SPON2 mRNA expression in three cell lines. (c) SPON2 protein expression in three cell lines. (d) The protein levels of SPON2 in Caki-1cells after knockdown by siRNA-SPON2 were detected by western blot.

SPON2 mRNA was significantly upregulated in ccRCC tissues, whereas normal tissues had low or no expression of SPON2 according to TCGA dataset (Figure 1(a)), which was confirmed in GSE53757 (Figure 1(b)). Among 94 matched-pairs of clinic samples (T and N), SPON2 mRNA expression was also significantly higher (1.70-fold) in ccRCC tissues than that in the adjacent normal tissues (*P* < 0.001). Based on immunohistochemical staining (Figures 2(a)), 49 of 94 (52.13%) ccRCC tissues exhibited positive SPON2 staining, whereas 22 of 94 (23.40%) normal tissues exhibited positive SPON2 staining (*P* < 0.05) (Table 1).

As shown in Figure 2(b), SPON2 mRNA expression was significantly higher in ccRCC cell lines Caki-1 (70.72-fold) compared to HK-2 cells, whereas no significant difference of SPON2 mRNA between 786-O and HK-2 cells. These results were also confirmed by western blot analysis (Figure 2(c)). Thus, Caki-1 cell was selected to investigate the role of SPON2 in ccRCC by in vitro assays. After transfection with three siRNA-SPON2 for 24 h, the significant reduction of SPON2 protein was observed in all cells, indicating that SPON2 knockdown was successful. Furthermore, the knockdown effect was best using siRNA-SPON2 (designated #1), which was used for further experiments (Figure 2(d)).

### 3.2. SPON2 Expression Is Correlated with Clinicopathological Characteristics

**Figure 3:**
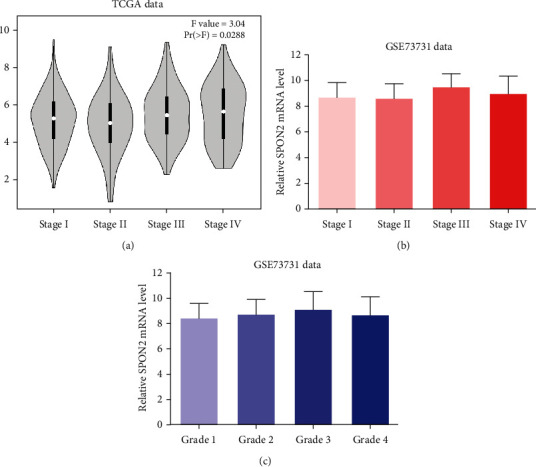
The correlation of SPON2 mRNA expression with the stage and grade. (a) The ccRCC patients with different stage from TCGA dataset. (b) The ccRCC patients with different stage from GSE73731. (c) The ccRCC patients with different grade from GSE73731.

As shown in Table 2, no significant association between SPON2 expression and patient age or gender was identified. SPON2 expression at protein and mRNA levels in patients with localized ccRCC was significantly correlated with TNM stage and Fuhrman grade (all *P* < 0.05). Analysis of TCGA data confirmed this result, which showed SPON2 expression was significantly correlated with TNM stage (Figure 3(a)). These findings were consistent with the analysis of GSE73731 (Figures 3(b)–3(c)).

### 3.3. SPON2 Expression Is Correlated with Prognosis of ccRCC Patients

**Figure 4:**
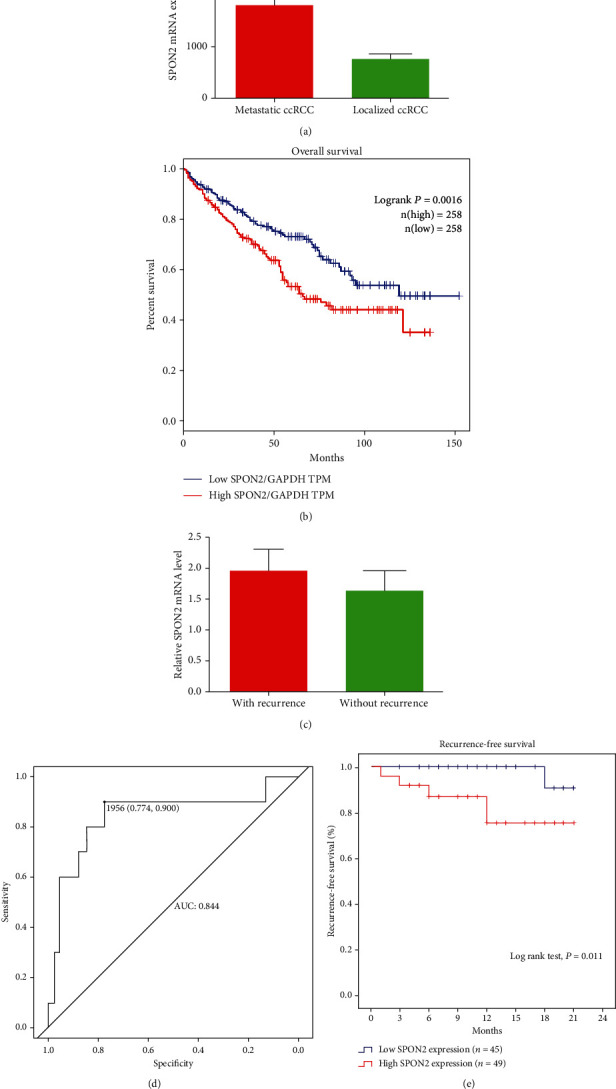
The correlation of SPON2 mRNA expression with prognosis. (a) SPON2 mRNA expression in patients with metastatic and localized ccRCC from GSE47352. (b) Kaplan-Meier overall survival analysis in patients from TCGA ccRCC dataset. (c) SPON2 mRNA expression in localized ccRCC patients with and without recurrence. (d) ROC analysis of SPON2 mRNA expression in localized ccRCC patients. (e) Kaplan-Meier recurrence-free survival analysis in localized ccRCC patients.

The data of GSE47352 confirmed that SPON2 expression in patients with metastatic ccRCC was significantly higher compared to patients with localized ccRCC (Figure 4(a)). Based on TCGA data, ccRCC patients with high SPON2 mRNA had a significantly worse overall survival (OS) probability than those with low SPON2 mRNA (Log-rank test, *P* = 0.0016; Figure 4(b)).

Among 94 patients with localized ccRCC, 10 patients had recurrence after nephrectomy. Patients with localized ccRCC presented recurrence had significantly higher SPON2 expression than those without recurrence (*P* = 0.001; *P* = 0.001, Table 2; Figure 4(c)). As shown in Figure 4(d), patients with localized ccRCC that presented recurrence could be predicted with a sensitivity of 90.0% at specificity of 77.4% (AUC = 0.844, 95% CI, 0.686−1.000) by SPON2 mRNA (cut − off value = 1.956). The RFS curve indicated that the high SPON2 expression group had a significantly higher recurrence rate compared to low SPON2 expression group (Log-rank test, *P* = 0.011; Figure 4(e)). The results of univariate and multivariate analyses for RFS are shown in Table 3. Univariate analysis indicated that tumor grade, sarcomatoid, SPON2 protein, and SPON2 mRNA (all *P* < 0.05) were significantly associated with RFS. By multivariate analysis, SPON2 mRNA (*P* = 0.012), tumor grade (*P* = 0.001), and sarcomatoid (*P* < 0.001) could be recognized as an independent indicator for RFS of patients with localized ccRCC after nephrectomy.

The C indices were 0.771, 0.706, and 0.915, respectively, when RFS was assessed with tumor grade, sarcomatoid, and SPON2 mRNA alone. Interestingly, C indices of tumor grade and sarcomatoid were improved to 0.893 and 0.915 with supplement of SPON2 mRNA. C indices were significantly improved to 0.939 with combination of all factors. Furthermore, all AIC values were decreased after supplement of SPON2 mRNA (Table 4).

### 3.4. SPON2 Knockdown Inhibits Cell Migration and Invasion In Vitro

**Figure 5:**
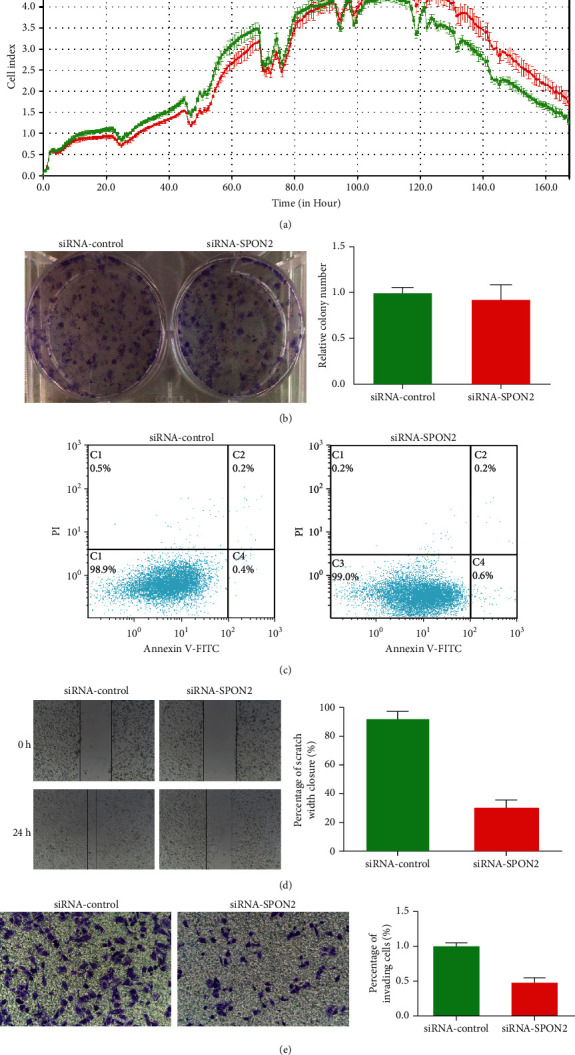
SPON2 knockdown on cell proliferation, apoptosis, migration, and invasion *in vitro*. (a) The cell growth curves of siRNA-SPON2 group and siRNA-control group. (b) Colony formation assay. (c) Apoptotic and early apoptotic fraction. (d) Wound healing assay. (e) Matrigel invasion assay.

According to the cell growth curves, no significant difference was found between siRNA-SPON2 group and siRNA-control group (Figure 5(a)). Colony formation assay showed that SPON2 silencing could not significantly inhibited colony formation (Figure 5(b)). As shown in Figure 5(c), the rate of apoptotic and early apoptotic fraction in siRNA-SPON2 group showed almost no variation compared to siRNA-control group. The wound healing assay showed that SPON2 silencing could significantly inhibit cell wound healing activity (Figure 5(d)). The matrigel invasion assay indicated that the number of invading cells was significantly decreased in siRNA-SPON2 group compared with those in siRNA-control group (Figure 5(e)).

## 4. Discussion

As a secreted ECM protein, SPON2 has multiple functions such as recruitment of inflammatory cells and activation of the innate immune response [18, 19]. Recently, SPON2 overexpression was found in numerous tumors including hepatocellular carcinoma [20], colorectal cancer [21], gastric cancer [22], prostate cancer [23], ovarian cancer [24], pancreatic cancer [25], and pulmonary adenocarcinoma [12].

In the present study, we used the TCGA and GSE datasets to show that SPON2 expression is significantly higher in ccRCC tissues compared with those in matched paratumorous tissues. This result was verified by qRT-PCR and IHC analysis of 94 paired ccRCC and matched normal tissue samples from our local patient population. We also found that SPON2 expression was significantly associated with TNM stage and Fuhrman grade.

Due to widely use of diagnostic cross-sectional imaging techniques, more ccRCCs were detected accidentally and diagnosed as the localized ccRCC [26]. Despite apparent complete surgical resection, 11% of patients with localized ccRCC also showed local or distant metastatic disease recurrence in this study. Compared to the patients without recurrence, the patients with recurrence had significantly higher SPON2 expression. Additionally, our results indicated SPON2 expression could be used to stratifying patients as either low or high risk for recurrence, and the patients with high SPON2 expression had poorer RFS than those with low SPON2 expression. Tumor size and stage are the important prognostic factors [27]. However, the multivariate Cox model analysis indicated that tumor size and stage were not independent risk factors for RFS in patients with localized ccRCC. This result could be explained by the fact that most of patients with localized ccRCC had the small, low stage tumors in this study. According to multivariate Cox model, high grade, sarcomatoid, and SPON2 overexpression were independent risk factors for poor RFS in patients with localized ccRCC.

Our research showed that SPON2 mRNA and protein levels in cell line Caki-1 (metastatic) were significantly higher compared with those of 786-O (nonmetastatic) and HK-2 (nonmalignant). However, no significant difference of SPON2 expression was found between 786-O and HK-2. Additionally, knockdown of SPON2 with siRNAs could not significantly change the proliferation, colony formation, and apoptosis of Caki-1 cells *in vitro*. This result was not consistent with previously reported results [9, 22]. We considered that SPON2 on tumor proliferation was not the same in different tumor types. This is the possible reason why no significant difference of SPON2 expression was found between ccRCC patients with stage I and those with stage II. In the current study, we found that SPON2 overexpression correlated with high risk of metastasis and recurrence in ccRCC. SPON2 silencing significantly reduced the invasion and migration ability of Caki-1 cells. However, Zhang et al. [20] reported that overexpression of SPON2 inhibited the migration and invasion abilities of hepatocellular carcinoma cell lines. This conflicting finding suggests that the function of SPON2 in cell invasion and migration is complex and needs further investigation.

The present study was associated with some limitations. It was a retrospective observational study and enrolled a relatively small number of patients; thus, the results may not be representative of other ccRCC populations. Because of the relatively short follow-up, the association between SPON2 expression and OS was not investigated in patients with localized ccRCC. The studies of SPON2 function *in vivo* and precise mechanism of SPON2 in ccRCC are required.

## 5. Conclusion

In conclusion, this study indicated that SPON2 was overexpressed in ccRCC and associated with tumor stage, Fuhrman grade, and recurrence after surgery in patients with localized ccRCC. SPON2 overexpression in ccRCC tissues is significantly correlated with reduced DFS following curative resection. Knocking down SPON2 resulted in suppressed cell invasion and migration *in vitro*.

## Figures and Tables

**Table 1:** The expression of SPON2 protein in ccRCC tissue and paired adjacent normal tissue.

Type of tissue	No.	SPON2 immunostaining (%)		
		—	1+	2+
ccRCC tissue	94	45	43	6
Adjacent normal tissue	94	72	20	2

**Table 2:** The relationship of SPON2 with the clinicopathological characteristics in patients with localized ccRCC.

Characteristics	No.	SPON2 mRNA (fold-T/N)	*P* value	SPON2 protein			*P* value
				0	1+	2+	
Age (years)			0.998				0.137
<60	60	1.70 ± 0.29		27	31	2	
≥60	34	1.70 ± 0.34		18	12	4	
Gender			0.756				0.210
Male	56	1.69 ± 0.31		25	29	2	
Female	38	1.72 ± 0.30		20	14	4	
Tumor diameter (cm)			0.593				0.735
≤4	58	1.69 ± 0.31		27	28	3	
>4	36	1.72 ± 0.30		18	15	3	
TNM stage			0.027				0.020
I	88	1.69 ± 0.30		43	41	4	
II	6	1.97 ± 0.29		2	2	2	
Fuhrman grade			0.003				0.001
G1+G2	90	1.68 ± 0.30		45	41	4	
G3+G4	4	2.14 ± 0.20		0	2	2	
Necrosis			0.471				0.623
Absent	82	1.71 ± 0.32		39	37	6	
Present	12	1.64 ± 0.22		6	6	0	
Sarcomatoid			0.069				0.057
Absent	86	1.69 ± 0.30		43	39	4	
Present	8	1.89 ± 0.35		2	4	2	
Recurrence (after surgery)			0.001				0.001
Absent	84	1.67 ± 0.49		44	37	3	
Present	10	2.03 ± 0.51		1	6	3	

**Table 3:** Uni- and multivariate analyses for predicting recurrence-free survival rate of patients with localized ccRCC.

Characteristics	Univariate analysis		Multivariate analysis	
	Hazard ratio (95% confidence interval)	*P* value	Hazard ratio (95% confidence interval)	*P* value
Age (<60 vs. ≥60)	3.817 (0.986-14.771)	0.052	—	—
Gender (female vs. male)	1.001 (0.282-3.549)	0.998	—	—
Tumor diameter (≤4 vs. >4)	1.364 (0.392-4.742)	0.625	—	—
TNM stage (I vs. II)	2.832 (1.485-9.523)	0.179	—	—
Tumor grade (1/2 vs. 3/4)	26.651 (7.081-100.313)	<0.001	14.506 (2.927-71.890)	0.001
Necrosis	0.468 (0.058-3.752)	0.475	—	—
Sarcomatoid	10.783 (3.117-37.306)	<0.001	24.145 (4.312-135.215)	<0.001
SPON2 protein	4.514 (1.736-11.741)	0.002	0.402 (0.076-2.126)	0.284
SPON2 mRNA	29.104 (3.672-230.684)	0.001	61.060 (2.471-1508.739)	0.012

**Table 4:** Comparison of the prognostic accuracy of the prognostic models and SPON2 expression for recurrence-free survival of patients with localized ccRCC.

Model	Recurrence-free survival	
	C-index	AIC
Tumor grade	0.771	66.285
Sarcomatoid	0.706	72.084
SPON2 mRNA	0.915	69.002
Tumor grade + Sarcomatoid	0.877	60.585
Tumor grade + SPON2 mRNA	0.893	60.176
Sarcomatoid + SPON2 mRNA	0.915	62.519
Tumor grade + Sarcomatoid + SPON2 mRNA	0.939	56.465

C-index: Harrell concordance index; AIC: Akaike information criteria.

## Data Availability

The data used to support the findings of this study are downloaded from TCGA database and GEO database. Three ccRCC mRNA expression datasets (GSE53757, GSE73731, and GSE47352) were obtained from the Gene Expression Omnibus (GEO) database (http://www.ncbi.nlm.nih.gov/geo/). TCGA-KIRC and corresponding clinical data used in this study were downloaded from The Cancer Genome Atlas (TCGA) data portal (https://gdcportal.nci.nih.gov/).
